# Prehospital Care Post-Road-Crash: A Systematic Review of the Literature

**DOI:** 10.1017/S1049023X25000202

**Published:** 2025-04

**Authors:** Joseph Cuthbertson, Greg Drummond

**Affiliations:** 1. University of Notre Dame Australia, School of Medicine, Fremantle, Western Australia; 2. Monash University Disaster Resilience Initiative, Melbourne, Victoria, Australia; 3. Fire and Rescue New South Wales, Australia; 4. Graduate School of Policing and Security, Charles Sturt University, Australia

**Keywords:** ambulance response, prehospital, road crash

## Abstract

**Objective:**

The aim of this study was to systematically review evidence that supports best practice post-crash response emergency care.

**Study Design:**

The research questions to achieve the study objective were developed using the Patient, Intervention, Control, Outcome standard following which a systematic literature review (SLR) of research related to prehospital post-road-crash was conducted according to Preferred Reporting Items for Systematic Reviews and Meta-Analyses (PRISMA) guidelines.

**Results:**

A total of 89 papers were included in the analysis, presented according to the PRISMA guidelines.

**Conclusions:**

This research explored and identified key insights related to emergency care post-road-crash response. The findings showed that interservice coordination and shared understanding of roles was recommended. Application of traditional practice of the “Golden Hour” has been explored and contested as a standard for all care. Notwithstanding this, timeliness of provision of care remains important to certain patient groups suffering certain injury types and is supported as part of a trauma system approach for patient care.

## Introduction

World-wide, more than one million lives are lost each year to road-traffic crashes, with up to 50 million people suffering injury, many of which are some of the most vulnerable.^1^ The World Health Organization’s (WHO; Geneva, Switzerland) Sustainable Development Goal Target 3.6 is to halve the number of global injuries from road-traffic accidents.^2^ Road trauma is already in the top ten (10) leading causes of death world-wide and is predicted to be the seventh leading cause of death by 2030.^1^ Several national and international strategies have been implemented to address this trend; the fifth pillar of the WHO Global Plan for the Decade of Action for Road Safety 2011-2020 road safety strategy (post-crash response) recommends: Increase responsiveness to post-crash emergencies and improve the ability of health and other systems to provide appropriate emergency treatment and longer-term rehabilitation for crash victims.^3^ This includes improving post-crash care by ensuring access to timely prehospital care and improving the quality of both prehospital and hospital care.^1^ Avoidable death related to road-crash incidents has been associated with injury secondary to poor prehospital care. This underpins the need for urgent action on the fifth pillar of the Global Plan for the Decade of Action for Road Safety 2021-2030 and improving post-crash response.^4^ Pillar five of the Global Plan seeks to improve emergency response and care to road crashes, and the subsequent injury rehabilitation, provision of mental health care, insurance and legal support, and data collection of road crash and injury.^5,6^ In recognition of the seriousness of this issue, nations such as Australia, Canada, and the United States of America have implemented public education and awareness campaigns to address this trend. While such campaigns in high-income nations are often complimented by comparatively well-funded and resourced prehospital care services, this is not consistent internationally.

Complicating matters, determining the most appropriate rescue techniques varies on the intended outcomes, which are influenced by the cultures of the organizations that are responsible for road trauma response, extrication, and patient treatment.^7,8^ Indeed, the WHO has reported that despite advancements in road safety, significant rates of avoidable death related to road trauma continue.^5^

To improve current approaches, services must first understand the current state of knowledge and review the lessons of the past. In the context of road-trauma response, specifically prehospital interventions in road-crash response incidents, this includes not only reviewing the insights gained during the Decade of Action for Road Safety, but also advances made since. This was achieved through application of a systematic literature review (SLR), a means of evaluating and interpreting all available research relevant to a particular topic area using a trustworthy, rigorous, reproducible, and auditable methodology.^9^ The aim of this study was to systematically review evidence that supports best practice prehospital post-crash response. This research was informed by multi-disciplinary international recommendations and practice. The research questions investigated included:

1. What evidence supports prehospital interventions during post-crash response?
2. How can identified trends support improved patient outcomes? And
3. Did the Decade of Action for Road Safety improve prehospital road-trauma response? If not, why not?

The paper is subsequently structured as follows. Firstly, a description is provided of the method used in the SLR. Following this, the results are presented according to Preferred Reporting Items for Systematic Reviews and Meta-Analyses (PRISMA) guidelines.^4^ The results and findings are then discussed and their implications for international road-crash response. The limitations of the study are acknowledged, and finally, the conclusions are presented.

Application of the findings from this study not only extend to facilitating improved practice in each of the themes examined, but they also provide a basis to assist future research endeavors and to contribute to the literature supporting international road-crash response.

## Methods

The research questions were developed using the Patient, Intervention, Control, Outcome standard to frame the search strategy (Table 1). The study consists of a systematic review completed in accordance with PRISMA principles.^10^

**Table 1.** Patient, Intervention, Control, Outcome

Patient	Road Crash Injured Patient of Any Age
Intervention	Extrication AND Prehospital Care
Control	No Extrication OR Prehospital Care
Outcome	No Worsening of Patient Injury

### Literature Search Methods

*Inclusion Criteria—*This study included English-language papers published from 2003 through 2024. Keywords describing road-crash response and emergency care were applied in the search strategy shown in Table 2, inclusive of peer-reviewed statistical studies/reports detailing post-crash response, as well as consensus guidelines, protocols, or other policy statements related to management of crash response by government and non-government organizations published from 2003-2024. The search strategy utilized in this study was structured to include a breath of literature that informed prehospital care in road-crash response and was further informed by a secondary hand search of bibliographies of identified papers. Review of title and abstract refined the selection for inclusion in final, full-text review.^11^

**Table 2.** Search Terms

Sources	Medline/PubMed, CINAHL Plus, ResearchGate, Google Scholar
Search Terms	Road Trauma *OR* Post Crash Response *OR* Road Crash Rescue *OR* Traffic Accident *OR* Traffic Crash *OR* Vehicle Rescue *OR* Transport Crash OR Transport Accident OR Passenger Crash OR Passenger Accident Emergency Medical Services OR Prehospital Emergency Care OR Fire and Rescue Mass Casualty Incidents
Limits	English Language AND Published from 2003-2024

*Exclusion Criteria—*Non-English-speaking literature, abstracts, citations, thesis, unverified or unsubstantiated press or news media reports, and articles that are not related to prehospital management of post-crash rescue patients were excluded.

### Quality Assessment

Two review authors independently assessed all included studies for risk of bias; any disagreement was resolved by discussion. The quality of the evidence was classified into four categories according to the Grading of Recommendations Assessment, Development, and Evaluation (GRADE) approach.^12^

### Publication Currency

The date range applied in this study was informed by previous research findings showing that extended date ranges provide little additional benefit.^13,14^ Seminal papers from outside the date range were considered for inclusion on consensus agreement by the authors; however, none were identified in either the hand searching or the review of the bibliographies and included studies.

## Results

In the identification phase of the review, the initial search strategy of databases yielded 17,041 studies for potential inclusion. Manual searching and a secondary search of bibliographies identified a further 90 studies for inclusion, providing a total of 17,131 studies.

An initial screening phase of title review was conducted by the two authors, with those either not meeting the full search criteria or outside of the defined scope excluded. A study was included for further review if initial screening could not confirm exclusion following review of the title. Overall, 17,020 titles were excluded during this process; in total, 111 studies progressed to the review stage.

**Figure 1.**
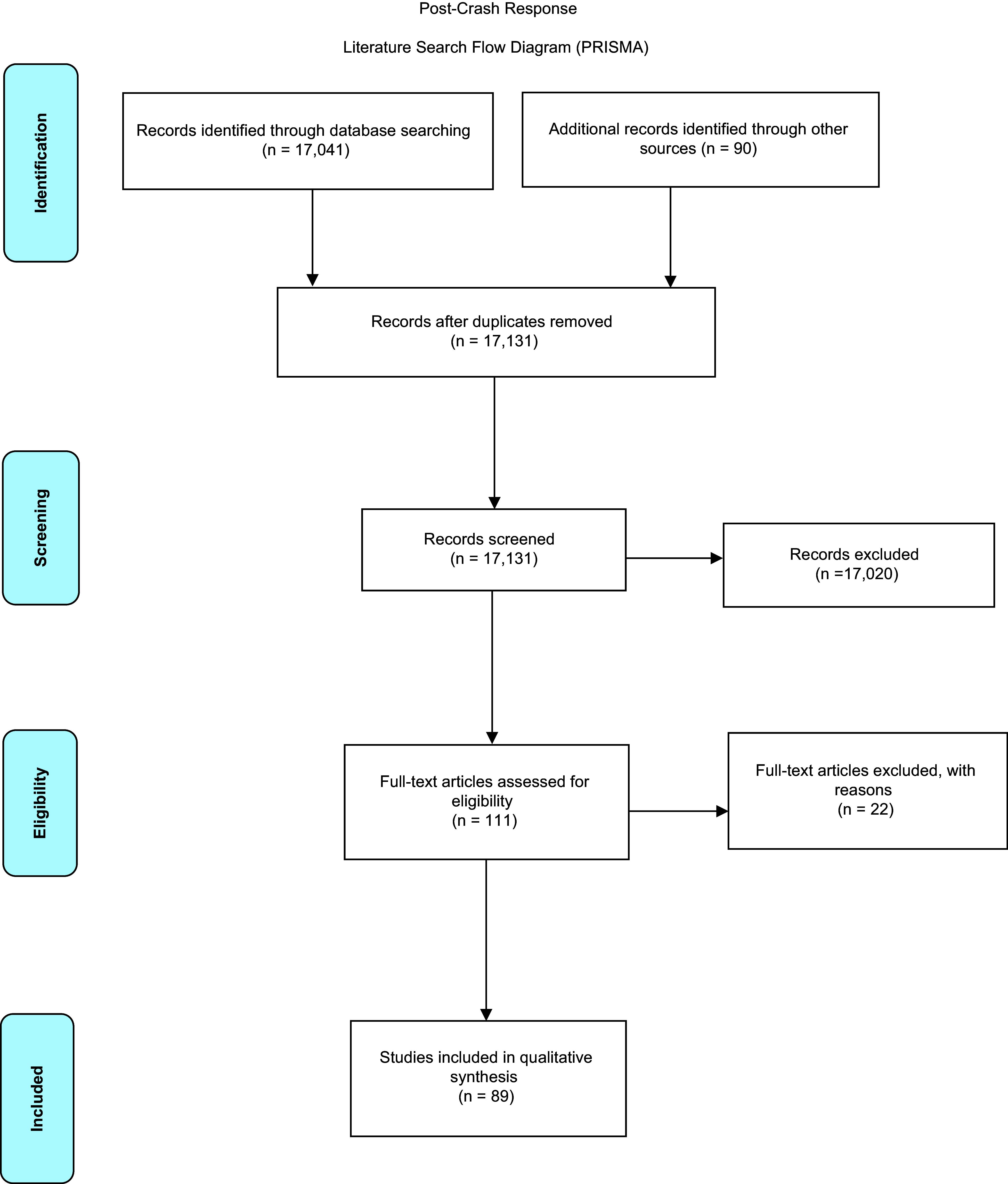
Literature Search Flow Diagram (PRISMA).

During the eligibility review, the authors initially completed a full-text review of the abstracts of the remaining 111 studies. Studies not meeting the full search criteria, or outside of the defined scope, were excluded (n = 22), resulting in a total of 89 papers that were included in the analysis. Any disagreement was resolved by discussion between the authors. Results are presented according to the PRISMA checklist (Supplementary Material; available online only) and demonstrated on the literature search flow diagram (Figure 1).

Quantitative analysis was not performed due to the heterogeneity of the research found in the systematic review, as the data were composed of different data types, structures, formats, or sources. The overall quality of papers utilizing GRADE criteria was found to be low with only three papers assessed as meeting high quality and four meeting moderate quality. This is possibly due to a large number of published case study reports.

Grounded theory process was used to identify themes from the collective literature. Narrative synthesis of findings was subsequently applied to explain the identified themes as it has proven useful for providing a comprehensive picture of the subject matter in question to guide new findings and conclusions.^15–17^ The results predominantly found peer-reviewed literature on “time” as a determinant of outcome, coordinated post-crash response, and post-crash response treatment systems (Table 3).

**Table 3.** Domains of Post-Crash Response Emergency Care

Domain	Published Articles
Time as a Determinant of Outcome	n = 28
Coordinated Post-Crash Response	n = 25
Prehospital Post-Crash Response Treatment Systems	n = 36

## Discussion

This study identified the following domains of prehospital post-crash response emergency care:

1. Time as a determinant of outcome;
2. Coordinated post-crash response; and
3. Prehospital post-crash response treatment systems.

### Domain 1. Time as a Determinant of Outcome

Multiple international studies have described time dependency as a function of road-crash response,^18^ specifically time to scene rather than time on scene. Byrne, et al found that longer response times were associated with higher rates of mortality post-motor-vehicle accident in both rural and urban settings.^19^ In an analysis conducted by Lee, et al investigating Fatal Traffic Crash-Reporting and Reporting-Arrival Time Intervals of Emergency Medical Services (EMS), it was found that whilst 90% of fatal crashes were reported to EMS within ten minutes, average reporting time in rural areas was greater than that in urban areas, and importantly, freeways required longer time for EMS arrival (8.3 minutes) compared with conventional road (6.8 minutes).^20^ Further to this, a retrospective review of over 1,400 road accidents in Spain by Sánchez-Mangas, et al reported that ten-minutes reduction in medical response is associated with a decrease in probability of death by one-third.^21^

### Domain 2. Coordinated Post-Crash Response

A consistent finding of this review was recommendation of improved inter-agency coordination and communication, with a focus on the establishment of unified command in response to mass-casualty accidents.^22–25^ Sadat, et al investigated barriers and facilitators to improve response to mass-casualty traffic accidents in Iran finding that improved coordination of agencies under a unified command, resource, and response management were desirable attributes. Whilst Sadat, et al undertook a qualitative study limited to one country, Sadat’s findings are consistent with investigations in other nations.^26^ Some systems have pre-determined criteria for response. Lee reported disaster medical team automated dispatch to road crashes where situations/incidents where more than ten casualties have already occurred, and additional casualties are suspected, and/or confirmation of multiple traffic accidents in vehicles over ten cars.^27^ Further to automated activation, Lee, et al also recommended a coordinated approach, joint operational procedures, and shared training to enhance effective scene communication in response to multi-vehicle collision in Korea.^27^ Whilst structural processes exist to enable early activation and deployment of resources to significant road-crash incidents, Lee found a lack of coordinated approach existed across agencies responding to the case study investigated.^27^

### Domain 3. Prehospital Post-Crash Response Treatment Systems

Understanding accident patterns can improve post-crash response training and readiness. Dong, et al identified side impact intrusion and secondary injury incidence related to patients not wearing seatbelts as key considerations for road-crash response practice in China.^28^ Mengistu, et al investigated road-crash mortality in Addis Ababa, Ethiopia with a focus on the relationship between prehospital care and mortality. Key findings from this retrospective study noted that the mortality of patients who did not receive prehospital care to road-crash response was three-times more likely in the following 24-hour period.^29^ Proposed timeframes of “Platinum 10 Minutes” and “Golden Hour” were not achieved in the majority of patients suffering road crash.^29^ This finding is reflective of the challenge in defining injury patterns in road crash that have time dependency to hospital for improved outcomes;^30,31^ in addition, further studies have noted that quality of care is also associated with survival, along with identification of time-dependent injury patterns.^5^

Traditionally, EMS has predominantly focused on response time; however, holistic feedback from first responders in understanding barriers and challenges in post-crash response may identify opportunities for improvement.^32,33^ Eftekhari, et al undertook such investigation finding that poor management of the crash scene, lack of adequate rules and regulation, poor management of time, low quality of training, poor communication and coordination, and low quality of victim management as key areas for improvement.^34^ These findings, whilst specific to one country, provide deeper insight into post-crash response quality improvement focus areas.

Trauma center establishment and incorporation into road-crash response practice was identified by Zarei, et al who reviewed modern concepts of system design to link key elements of response and health capacities that, when coordinated, can deliver improved outcomes.^35^ Contemporary practice in road-crash response supports a concept of operations where decision making and health system design is informed by patient acuity, transport mode and time, and destination according to treating facility capability (ie, trauma center), an approach supported by multiple researchers investigating trauma transport practice.^35–41^

Whilst some studies have reported an association between prehospital time and trauma survival,^42^ there is a lack of high-quality evidence supporting the “Golden Hour” theory and evidence showing a lack of associated outcomes in road-crash response.^29,43–45^ Further to this, the application of Basic Life Support (BLS) and Advanced Life Support (ALS) interventions as part of post-crash response needs to be critically reviewed.^46–48^ Whilst Noland has reported an association between improvements in medical care and reduced crash fatality,^49–51^ Al-Shaqsi reported that “existing studies reporting potential benefits of Advanced Life Support were descriptive studies with small sample sizes as opposed to testing of hypothesis and tended to be grossly confounded and biased.”^47^ At the same time, Jayaraman, et al in their systematic review reported no evidence to suggest that ALS training for ambulance personnel improved the outcomes for injured people.^46^ Likewise, Lydon, et al reported that “… it is unclear if improvements to post-crash response can deliver significant benefits.”^52^

McDermott, et al investigated 243 road crash cases in Victoria, Australia from 1997-1998 that were reviewed by multi-disciplinary medical, forensic, and prehospital expert panels to evaluate prehospital treatment during road trauma.^53^ It found that 77% of patients had prehospital errors or inadequacies, of which 67% contributed to death; however, technique errors and diagnostic delays were infrequent. A single death was considered preventable, with another two being potentially preventable. Key points from the study included:

- 21% of all fatalities required extrication, with a greater percentage requiring extrication in regional areas.
- 48 technique errors were identified, of which 33 contributed to death. Most frequent errors were failed intubation and failed intravenous access.
- 10 of the 18 diagnosis errors contributed to death. This included tension pneumothorax (1); hypovolemic shock (2); misplaced endotracheal tube (1); fractured ribs/bilateral flail chest and respiratory failure (2); severe hypoxia treated with morphine, not O2 (1); fractured pelvis (1); and under-estimation of severity of injury (2). And,
- Load-and-Go protocols should be implemented for patients not trapped, which allows for most prehospital interventions to be commenced/completed enroute.^53^

The study concluded that since the review of 1998 data, the high prevalence of prehospital deficiencies had been addressed by a Ministerial Task Force on Trauma and Emergency Services. However, the study did not provide information, discussion, or further analysis to explain or support this conclusion. It should also be noted that while McDermott, et al technically met the inclusion criteria of this current SLR as it was published in 2005, the cases it reviewed were from the late 1990s, well before the Decade of Action for Road Safety.

In this study, three specific research questions were explored:

1. What is the evidence to support post-crash response practices?
2. How can identified trends support improved patient outcomes? And
3. Did the Decade of Action for Road Safety improve prehospital road-trauma response? If not, why not?

With respect to the first question, 89 studies met the inclusion criteria for the study of 17,052 potential studies initially identified from four major databases. Whilst there was limited commonality between included studies, this shows compelling evidence to inform contemporary post-crash response practices.

With respect to the second question, again, there were limited trends to support improved patient outcomes. Interestingly, there was no evidence to support the time-honored concepts of the “Platinum 10 Minutes” and the “Golden Hour,” with the limited evidence located appearing to question the validity of these concepts.^30,43^ The single study that focused on scene management suggests that good rescue and treatment practices may be more important than whether a patient reaches a trauma center within a defined or arbitrary time frame.^28^ Further to this, the evidence suggests that BLS may be a more appropriate intervention when responding to road-crash trauma than ALS practices.^46–48^ Indeed, the findings of Ma and Lee, et al may indicate time from crash to patient treatment on scene may be a more important factor than time to a trauma center.^18,20^ Ultimately, these findings suggest, albeit in a limited way, that there is little contemporary evidence to support post-crash response practices beyond those completed more than a decade ago by McDermott, et al.^53^ Further, these findings suggest that prehospital services should re-examine current practices and consider whether the funding and time invested in ALS training, on-going certification, and associated equipment would be better spent on additional prehospital ambulances and resources that would ultimately reduce the time to accessing/reaching dedicated trauma centers.

With respect to the third question, no evidence was found in the literature that the Decade of Road Safety Action has had an impact on improved patient outcomes or reduced road fatalities. A review of published statistics tells a similar story; in Australia, road-traffic deaths have remained relatively unchanged over the last decade, with a slight decrease in fatality rates of 5.1% to 4.6%, while in the United States, road-crash fatality rates have increased with a rate 2.3-times higher than the average of other high-income countries.^54,55^ During the Decade of Action on Road Safety in the period from 2013-2016, there was no reduction in road-traffic deaths in any of the low-income countries reviewed by the WHO.^4,56^ Similar trends were reported by WHO for countries within Southeast Asia from 2007-2015.^57^ Even with the limited improvements in prehospital care, the ever-increasing number of people relying on motor-vehicle transport, coupled with poor driving behaviors, unsafe vehicles, and sub-standard road conditions, has resulted in increased rates of road trauma globally.^1,55^ Part of the problem may be associated with the inability of governments and organizations to consider a systems approach to road-traffic safety as opposed to examining individual interventions. Indeed, Tavakkoli, et al suggest this may be a significant cause in the Decade of Action on Road Safety ultimately being ineffective.^56^

## Limitations

The studies of post-crash practice identified in this SLR were predominantly retrospective case reviews; there are limited prospective studies related to post-crash response practice. The studies that met inclusion criteria were heterogenous; quantitative analysis was undertaken.

Papers not in English language were an exclusion criterion; as such, papers not in English related to post-crash response were not included. However, they were identified as a valid source of global mortality data.

## Conclusion

The declaration of the Decade of Action for Road Safety 2011 to 2020 by the United Nations General Assembly sought to reduce road trauma and improve road-trauma response.^58^ This SLR identified pre-established joint agency planning, training, and coordination of response agencies involved were recommended in post-crash response. Traditional approaches of “Golden Hour” and “Platinum 10 Minutes” of care at post-crash scenes are rarely achieved in the reviewed literature and there is evidence to suggest they are not associated with improved outcomes in the context of civilian road-trauma response. Timeliness of provision of care remains of importance to certain patient groups suffering specific injury types, and trends in health system design that support a trauma system approach for patient care represent contemporary practice.

Following the Decade of Action for Road Safety, there has been little, if any, improvement in outcomes of prehospital response in multiple-casualty road-trauma incidents. It is posited that unless the foundations and guiding principles of prehospital trauma response are critically reviewed, the essential and limited available resources required to improve road-trauma treatment outcomes will continue to be sub-optimal, if not far from evidence-based best practice.

## Supporting information

Cuthbertson and Drummond supplementary material Cuthbertson and Drummond supplementary material

## Supplementary Material

To view supplementary material for this article, please visit https://doi.org/10.1017/S1049023X25000202
